# Student-centered case-based teaching and online–offline case discussion in postgraduate courses of computer science

**DOI:** 10.1186/s41239-022-00374-2

**Published:** 2023-01-31

**Authors:** Xinhong Zhang, Boyan Zhang, Fan Zhang

**Affiliations:** 1 School of Software, Henan University, Kaifeng, 475004 China; 2 Henan Key Laboratory of Big Data Analysis and Processing, Henan University, Kaifeng, 475004 China

**Keywords:** Case-based learning, Student-centered teaching, online–offline case discussion, Postgraduate teaching

## Abstract

This study explores a student-centered teaching method in postgraduate courses. Teacher-centered classroom teaching cannot fully stimulate learning initiative and enthusiasm of students. Student-centered means that students actively learn and construct knowledge by participating in teaching activities. This study presents a student-centered online–offline hybrid teaching method, which adopts student-centered case-based teaching and online–offline case discussion in the postgraduate courses of computer science. The latest engineering cases are integrated into teaching and a case library is constructed. Taking the digital image processing course as an example, student-centered teaching allows students to choose what to learn and how to learn. Case-based teaching makes students better understand the application of theory of knowledge. It can introduce multiple perspectives, promote understanding and reflection on problems, and help students develop higher-level thinking, analysis, and synthesis skills. This study explores online–offline case discussion method in the student-centered teaching and proposes the principles of case design of postgraduate courses. Revised Bloom’s taxonomy is used for teaching assessment. The actual teaching effect shows that student-centered case-based teaching and online–offline case discussion have achieved better teaching effect.

## Introduction

Teacher-centered classroom teaching is the main mode adopted in current postgraduate courses of computer science. However, this traditional teaching mode cannot fully stimulate the learning initiative and enthusiasm of students, and is also not conducive to cultivating their innovative thinking ability. We find it difficult to achieve the teaching goal by completely adopting teacher-centered classroom teaching for science and engineering graduate students, because their studies focus more on engineering and practical applications. So, it is necessary to explore a new teaching mode. Case-based teaching (CBT) or case-based learning (CBL) provides a solution to solve the problems above (Sangam et al., 2021).

In case-based teaching, a case is defined as a description based on a real event or situation in which sufficient detail is provided to assist students in the analysis and solution of problems (Prada et al., 2020; Tan et al., 2014). The development of information technology has created a variety of possibilities for the design of cases. Therefore, cases are also defined as the typical teaching events using multimedia formats, such as video, audio, pictures, animation, and web pages. The case-based teaching method guides students to carry out a series of learning activities, including analysis, discussion, problem-solving, evaluation, reflection etc., which is helpful for students to develop higher-level thinking, analytical and integrative skills (Tawfik et al., 2017; Strobel et al., 2013). Some studies have shown that case-based teaching makes up for the deficiency of passive acceptance of learning, and has a significant impact on promoting knowledge transfer and knowledge application.

This paper presents a student-centered online–offline hybrid teaching method for the postgraduate courses of computer science, which adopts case-based teaching and online–offline case discussion. The latest engineering cases are integrated into teaching and a case library is constructed. Taking the digital image processing course as an example, student-centered teaching allows students to choose what to learn and how to learn. Case-based teaching makes students better understand the application of theory. It can introduce multiple perspectives, promote understanding and reflection on problems, and help students develop higher-level thinking, analysis, and synthesis skills. Revised Bloom’s taxonomy is used for teaching assessment.

The main contributions of this study are as follows:

1. Exploring student-centered teaching in postgraduate courses.
2. Using cases as the main contents of teaching.
3. Adopting the case-based teaching method.
4. Exploring the online–offline case discussions in the student-centered teaching.
5. Proposing the principles of case design of postgraduate courses.

This paper is organized as follows: "Introduction" section deals with the introduction. "Literature review" section reviews the relevant literature. "Methods" section describes the method of case library construction and the method of student-centered case-based teaching. "Result" section provides the implementation results of our teaching method. "Discussion" section discusses this study. Finally, "Conclusion" section draws conclusion.

## Literature review

### Case-based teaching

The design and implementation of case-based teaching activities create opportunities for an exploratory new learning mode (Goeze et al., 2014). By participating in a series of activities in case-based teaching, students actively develop skills of knowledge application and problem-solving, and conduct abilities of higher-level thinking, analysis and synthesis (Newton et al., 2015b). Several studies have highlighted how case-based teaching enhances students’ comprehension and critical thinking skills (Leon et al., 2015). Students’ reflective and critical thinking skills are promoted as they work on cases that challenge them to deal with issues of multiple layers and complex dimensions.

With the development of information technology, the cases used for teaching have been transformed from the textual narration to the multimedia-based presentation. Multimedia cases are gradually applied to the online learning environment (Luo et al., 2018). Multimedia case teaching has its unique advantages. It can better simulate the complexity of real-world problems (Rico & Ertmer, 2015). For example, the cases presented by interactive multimedia can attract and motivate students, and can effectively promote knowledge transfer. Hewitt et al. use video cases as the carrier of case-based teaching. They encourage students to think, discuss, solve, and reflect the problem through pause and interaction of the video case at each key point. The final results prove that video case teaching promotes the learning interest and motivation of students (Hewitt et al., 2003). In the exploration of learning effects, Choi et al. use the interaction and feedback functions of multimedia cases to provide feedbacks of experts at each decision point of case problems. During the case learning process, students can view expert opinions to gain an in-depth understanding of the problem. The results of the learning effect evaluation demonstrate the effectiveness of multimedia case teaching (Choi & Lee, 2008). Research results show that multimedia case teaching improves learning motivation of students, helps students better master knowledge, and improves their problem-solving ability. Yoon et al. use learning analytics to gain useful insights into student learning in a video-based online learning environment (Yoon et al., 2021). Based on the observed patterns of log behavior, students can be divided into two categories: active learners and passive learners. Aactive learners have higher academic performance than passive learners.

When constructing teaching cases, teachers should start by identifying goals, identifying skills, and deciding which concepts students should learn. Through this process, teachers carefully consider the learning outcomes that students should achieve (Jevne et al., 2021). Newton et al. argue that case production can be either open-ended or guided by challenges or problems, depending on the teaching purpose and student population. The case should enhance students’ interest by using stories they can relate to (Newton et al., 2015a).

### Case discussion

Case discussion is an important part of case-based teaching. It is regarded as the key to the success of case-based teaching. Teachers guide students to express their personal opinions on the case, and realize the sharing process of problem exploration and knowledge construction (Ertmer & Koehler, 2014). Some studies have shown that case discussions can introduce a variety of viewpoints, promote students’ understanding and reflection on problems, and help students transfer and apply knowledge. In general, case discussion has a good role in promoting case-based teaching (Ertmer & Koehler, 2015). Yew et al. believe that students’ participation in the interactive case activities can help students to actively construct knowledge, improve learning interest and learning engagement, and enhance learning performance (Yew & Yong, 2014). The targeted guidance of teachers also improves the learning experience and learning effect (Long & Koehler, 2021; Kim, 2022; Roels et al., 2021; Zhang et al., 2019, 2022a). Lock et al. provide expert understandings of online discussions. These understandings address real-world issues related to diverse and digital classrooms (Lock & Redmond, 2021). Zhang et al. use epistemic network analysis (ENA) to explore the collaborative problem-solving processes of students and teachers in different online collaborative learning tasks (Zhang et al., 2022b). By investigating the academic performance of collaborative problem-solving patterns, they reveal in detail the relationship between cooperative problem-solving and students’ academic performance.

The online learning environment presents both opportunities and challenges for case discussions (Mcpartlan et al., 2021). Broadbent et al. evaluate whether self-regulated learning (SRL) impacts with students’ academic performance in both online and offline learning environments (Broadbent et al., 2021). Among students who study online, those who benefit the most are those who are confident, able to manage their time and discipline their efforts. Turk et al. believe that online course instructors should provide self-supporting goals, choices, guidance, and feedbacks. They should also ensure their effective interactions with students. The interactive learning environment for students to interact with their peers should be socially and emotionally trusting (Turk et al., 2022).

As an important activity of the case-based teaching method, online discussions create conditions for online teaching or online–offline hybrid teaching. online–offline hybrid teaching is a kind of teaching that combines online teaching with traditional teaching (Zhao et al., 2022; Yi, 2022; Peng & Wei, 2021). online–offline hybrid case discussion has special advantages. (1) online–offline hybrid case discussion breaks the limitation of time and space. It realizes a more flexible and free way of asynchronous discussion. Online case discussion prolongs the timeliness of classroom discussion and provides students with a more personalized learning pace, more flexible problem-solving and reflection space. (2) online–offline hybrid case discussion creates more favorable conditions for the participation of teachers and invited experts. Flexible online and offline interaction helps teachers to provide more accurate guidance and feedback, which makes it possible for highly interactive case teaching. However, the asynchronous discussion makes the problem discussion lose the characteristics of timely feedback, and the online discussion weakens the guiding role of teachers to a certain extent (Wu, 2022; Li et al., 2021).

### Student-centered teaching

The student-centered teaching concept reflects the principles of constructivism theory. Student-centered means that students actively learn and construct knowledge by participating in teaching activities (Zhienbayeva & Abdigapbarova, 2021; Mamnpoba, 2021). In student-centered teaching, the teaching method changes from teaching to guiding; The teaching subject changes from teacher to student; The teaching content changes from textbook to practice; The assessment method changes from traditional examinations to diversified procedural examinations. Student-centered teaching is closely related to students’ learning enthusiasm. Specifically, student-centered teaching can help students actively participate in learning and achieve better grades. When students’ needs are more comprehensively met, student attendance will increase, and the possibility of dropping out will be reduced. They will focus more on their studies and be better prepared for graduation. Constantinou et al. point out that student-centered teaching involves not only academic learning, but also other skills, such as active participation in society or community, professionalism, mental health, etc. Therefore, student-centered teaching requires a holistic view of the learning and development of students (Constantinou, 2020). The corona virus 2019 (COVID-19) global pandemic has forced higher education to transform to the online learning mode. This provides an opportunity to adopt student-centered teaching. Active learning can improve students’ performance and close the achievement gaps for underachievers (Sandrone et al., 2021). Angel et al. adopt the method of flipped classroom to carry out student-centered teaching (Mingorance Estrada et al., 2019). Compared with the traditional teaching, this method significantly improves student performance, increases student interaction, and improves classroom attendance and engagement. Teachers’ feedback and teacher-student interaction will effectively mobilize students’ learning enthusiasm. Moges et al. believe that in order to improve the teaching effect, teachers should innovate and diversify teaching methods to attract students to participate. In addition, teachers and students need to be properly trained. Both of them need to understand the impact of student-centered education so that they have a clear understanding of their roles and responsibilities (Moges, 2019).

Both the teacher-centered teaching method and the student-centered teaching method are useful. The best teaching method is comprehensive. Different teaching methods can learn from each other and complement each other. Several studies have revealed the value of combining traditional teaching with student-centered teaching. A way of combined approach is for students to try to solve problems on their own. The teacher then teaches the correct problem-solving steps and compares the student’s solution to a standard problem-solving solution. This model can be called learning before teaching. Exploratory learning is a teaching method of learning before teaching. Exploratory learning refers to the exploration of new problems by students before they are taught related concepts and solutions (Chung & Ho, 2021). The purpose of exploratory learning is to give students the opportunity to explore new topics for themselves before accepting traditional teaching (Weaver et al., 2018; Schalk et al., 2017). Another way of combining application is to teach the relevant knowledge and correct solution directly, and then ask the students to do problem-solving exercises using the method taught by the teacher. This model can be called teaching before learning. The most typical example of teaching before learning is problem-based learning (PBL). PBL is a teaching method that students are presented with a real or realistic problem, such as a case, and use inductive reasoning to learn both information about the topic and how to think critically about it. Through PBL, students can acquire both knowledge and skills of collaboration, communication, and reflection (Kapur, 2016; Armstrong et al., 2021).

### Self-regulated learning and Bloom’s taxonomy

Self-regulated learning (SRL) refers to the process by which students activate and maintain their own thoughts, feelings and behaviors, and systematically achieve learning goals (Song et al., 2021; Tran et al., 2022). Learning goal, efficacy and learning strategy are three important components of self-regulated learning (Granberg et al., 2021). The most striking feature of self-regulated learning is that students have actual control over their own learning. They can cognitive and control the processes directly to achieve their learning goals (Callan et al., 2021; Guo et al., 2021a; Tuti et al., 2021). Rovers et al. compare the validity of several different methods of self-regulated learning (Rovers et al., 2019). The self-reported questionnaire can reflect the overall level of students’ self-regulated learning. In contrast, behavioral measures provide more accurate explanations when students are asked to report specific self-regulated learning strategies. Many studies have shown that the external feedback from teachers could promote students’ self-regulated learning (Yunus et al., 2021; Aguilar et al., 2021). Teacher’s feedback and evaluation could increase the intrinsic motivation of students. For example, encouraging students to participate in more challenging tasks can improve the self-regulated learning level of students. Students typically exhibit more academic help-seeking behavior and make more efforts in response to teachers’ support (Guo et al., 2021b).

**Fig. 1:**
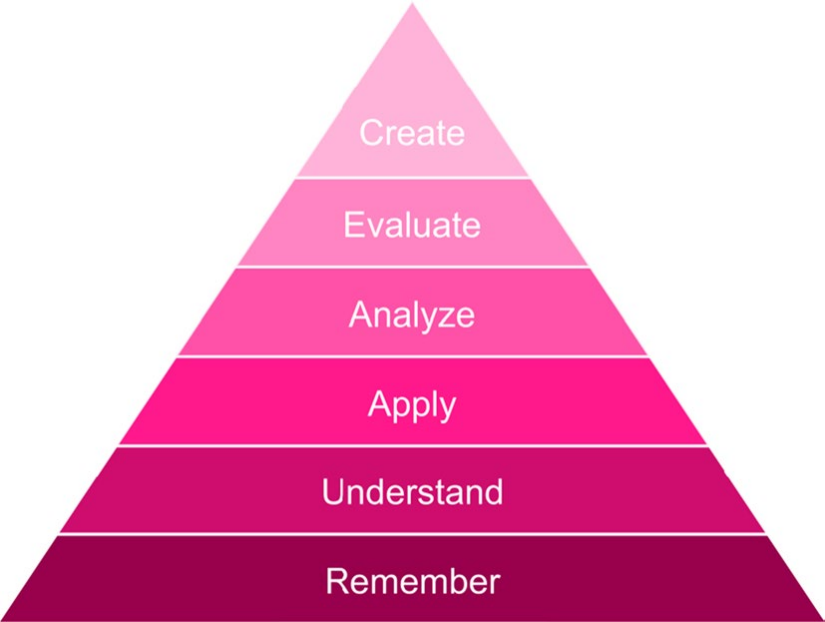
The revised Bloom’s taxonomy

Bloom’s taxonomy is a hierarchical model that divides learning into levels of complexity. The revised Bloom’s taxonomy divides the cognitive process dimensions in six levels (Krathwohl, 2002). The six levels from low to high are: Remember, Understand, Apply, Analyze, Evaluate, and Create. Figure 1 shows the revised Bloom’s taxonomy. Bloom’s taxonomy is a hierarchical model designating learning into levels of complexity and is often used to structure course experiences such as learning objectives, assessments, and pedagogical choices (Killion et al., 2022). Bloom’s taxonomy of educational goals reflects the relationship between knowledge learning and ability development through the structure of knowledge dimension and cognitive process dimension. It is also a tool for the evaluation of teaching objectives and the assessment of examinations (Vieyra & Gonzlez, 2020). Desha et al. propose a new model to assess the development of problem-solving skills based on Bloom’s taxonomy (Desha et al., 2021). They wonder how the design might have stimulated or dampened student appreciation of complexity, and how these findings aligned with desired expectations. To explore this, the learning materials are evaluated through Bloom’s taxonomy. The goal is to understand the extent to which the course content exposed students to the spectrum of problem-solving contexts. Dolan et al. propose some recommendations for the use of virtual simulations in the current learning environment by studying learning theories, learning styles, and Bloom’s revised taxonomy (Dolan et al., 2021). Synchronous debriefing with students, faculty, preceptors, and peers provides the opportunity for scaffolding to support students’ learning needs and foster reflection.

## Methods

### Construction of a case library for the digital image processing course

Digital image processing is a course for computer science graduate students. This course is both theoretical and practical. At present, the cases in the teaching materials are relatively outdated and cannot reflect the latest research progress. In addition, because there is no experiment arranged, students’ sense of participation is not strong. This paper explores the method of student-centered case-based teaching and online–offline case discussion during digital image processing.

The construction objectives of the case library (or case base) of digital image processing course are as follows. The design of case library is student-centered. The information and data in the case should fully consider correctness and reliability. Case library requires constant maintenance and updating. Cases should meet the requirements of typicality, objectivity, advancement, and innovation. Students can acquire the knowledge of image processing efficiently and quickly from cases. Through case-based teaching, students should be able to broaden their horizons, stimulate their learning interest and improve their practical abilities.

### Principles of case design

We propose the following principles of case design for the engineering courses:

1. Comprehensiveness. Multiple knowledge points are run through case-based teaching. Knowledge points are presented to students in the form of interconnected case applications. Students can discover and master knowledge in the practice process of problem-solving. Therefore, it is necessary to ensure comprehensive requirements in case design, so that the designed cases can be seamlessly connected with the knowledge points of the textbooks.
2. Advanced. The latest research results are collated into teaching cases to replace the outdated cases of the textbooks. The teaching case should be advanced and innovative. For example, choosing teaching cases using artificial intelligence (AI) and other new technologies can make up for the insufficient introduction of new knowledge and new technologies in the textbooks.
3. Engineering. The practical engineering problems are transformed into teaching cases to reflect the practicability of the digital image processing course. For example, we invite engineering experts from partner companies to write cases together. These cases place more emphasis on the combination of theory and practice.

### Case design and selection

The design and selection of cases should give students a solid understanding of the application and implementation of theories, methods, and models. Well-designed cases can guide students to discover, analyze, and solve problems. Cases should involve all the knowledge points and their applications of each chapter of the digital image processing course.

**Fig. 2:**
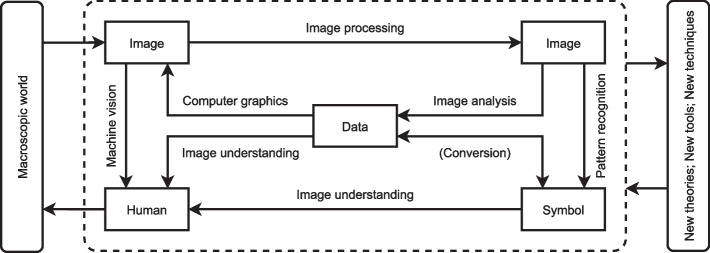
The relationship between a digital image processing course and other disciplines or courses

Digital image processing has been widely used in many fields. The applications of digital image processing are interconnected with many disciplines, such as mathematics, physics, biology, medicine, and computer science. At the same time, it is supported by many new theories, new tools, and new technologies. Artificial intelligence (AI) is the main application field of digital image processing. The digital image processing course intersects with many courses, such as pattern recognition, machine vision, computer graphics and other courses. Therefore, the design and selection of cases should avoid being limited to the knowledge points of a digital image processing course, but should try to reflect interdisciplinary characteristics and interdisciplinary integration. Figure 2 shows the relationship between a digital image processing course and other disciplines or courses.

According to the case design principles mentioned above, we designed and selected some teaching cases for the digital image processing course. In order to facilitate students’ extracurricular study and online–offline discussions, we provide the case study documents for each case. The contents of case study documents include preparatory knowledge, theoretical knowledge, technical points, implementation process, results, and demonstration programs. Source codes are also provided in most of the cases. Case study documents are distributed online for students to study and practice after class. Some of the case study documents of the digital image processing course are shown in Table 1.

**Table 1:** Some of the case study documents of the digital image processing course

Number	Name of case study documents	Number of pages
1	Color-Coded Imaging Technology	11
2	Lattice Boltzmann Method and Image Processing	29
3	Magnetic Resonance Imaging	21
4	Text Recognition of Images	19
5	Halftone and Inverse Halftone Technology	13
6	Pattern Recognition	21
7	Differential Homeomorphic Registration Algorithm	16
8	Phase Correlation Based Subpixel Image Registration	15
9	Zernike Moment Subpixel Edge Detection	15
10	Medical Image Processing Cases	13

### Arrangements of student-centered case-based teaching

Our student-centered case-based teaching process adopts an online–offline hybrid approach. The teaching arrangement includes theoretical knowledge lectures (offline), case introduction (offline or online), extracurricular literature reading, extracurricular experiments, and group discussions (online or offline).

1. Lectures on theoretical knowledge
  Classroom teaching is used to describe the knowledge background and the application fields of cases. Problems are elicited through cases, and theoretical concepts and knowledge points related to cases are explained. In the teaching of theoretical knowledge, we only teach selected contents of the textbook to save the limited classroom teaching time. The rest of the contents are left to students for self-study and discussion.
2. Introduction of cases
  When and how to introduce cases is also considered. According to the teaching plan, we determine which cases are introduced in which chapters, how each case is presented, and how long it takes to explain or demonstrate the case. The introduction of cases not only enables students to better understand the practical application of theoretical knowledge, but also enables these cases to effectively support the relevant knowledge points in the textbook.
3. Extracurricular literature reading and extracurricular experiments
  We arrange for students to consult the literature on the content of theoretical knowledge of the introduced cases. Students should run the source codes provided by case after class and improve it, or design new codes according to the requirements of the case and the theoretical knowledge they have learned. They need to implement the codes themselves to achieve the required functions of the case.
4. online–offline discussion
  online–offline group discussions are conducted on the theoretical knowledge lectures, introduced cases, and experimental results. We encourage students to ask questions and encourage their sense of innovation. When necessary, we also arrange for oral presentations by group representatives.

### Implementations of student-centered case-based teaching

**Fig. 3:**
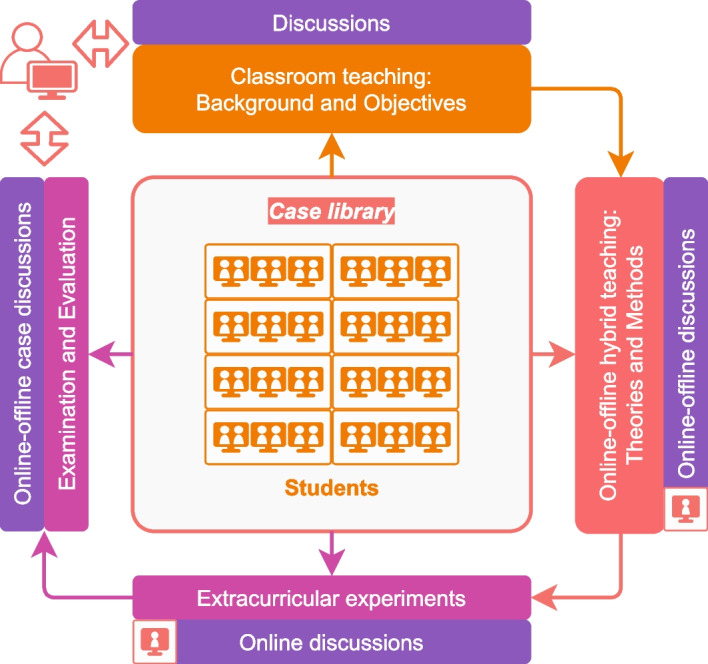
The implementations of student-centered case-based teaching for the digital image processing course

The case-based teaching process is designed as student-centered. The main teaching content is cases and textbooks. The implementations of student-centered case-based teaching for digital image processing course is shown in Fig. 3.

1. Providing case documents online and arranging students to preview before class.
2. Introducing the background and objectives of the case in traditional classroom teaching mode, and teaching relevant knowledge and theories. Classroom group discussions are arranged during this process.
3. Guiding students to explore cases in online and offline hybrid teaching mode. Students learn relevant theories and methods through case studies. Students are encouraged to come up with their own solutions based on the theories and methods they have learned. This is a learning-imitation-exploration-innovation process (innovation is optional). Online and offline discussions are arranged during this process.
4. Students implement extracurricular experiments based on the source codes provided by the case or design new codes by themselves. The analysis of the experimental results also needs to be done themselves. They can communicate about the problems they encountered, seek help, or discuss solutions of problems and experimental results through online or offline discussions.
5. Introducing, sharing, and demonstrating the learning results of the case in online or classroom teaching mode. Finally, we organize exams and evaluate the exam results.

### Assessment methods

The revised Bloom’s taxonomy is used for teaching objective evaluation and examination assessment. After the introduction of the case-based teaching mode, the assessment method also needs to be adjusted accordingly. We no longer only use the static indicators, such as exam scores, but introduce the dynamic indicators for the assessments, such as case study reports, experiment reports, literature reading reports, oral reports, and records of participation in online–offline group discussions. These assessments consider the characteristics of case-based teaching and realize individualized evaluation.

## Result

### Research object

This research is based on the digital image processing course for graduate students of computer science. Full-time postgraduate students of three consecutive years participated in this research. Since the number of graduate students varies from year to year, we randomly selected 100 students from each year as one group. All the students in the three groups are to study the digital image processing course for the first time, and they had never learned any cases used in this research before. The three groups of students are roughly equivalent in gender ratio, age distribution, and course-related prior knowledge. In addition, when the questionnaire of learning interest and learning motivation were scored, the feedbacks of the three groups of students are not significantly different. Which indicates that they have similar learning interests and learning motivations.

Different teaching methods, teaching contents, and assessment methods were adopted to the three groups of students. The differences between them are shown in Table 2.

**Table 2:** Different teaching methods, teaching contents, and assessment methods for the three groups

Group	Teaching method	Teaching contents	Assessment method	Feature
A	Classroom teaching	Textbook	Exam	Traditional teaching
B	Classroom teaching + self-regulated case learning	Textbook + cases	Exam (exam does not involve cases)	Traditional teaching
C	Online–offline hybrid teaching + online–offline case discussion	Cases + textbook	Exam + experiment reports + case study reports	Student-centered case-based teaching

### Student-centered case-based learning

The case-based teaching process is designed as student-centered. After the traditional classroom teaching for the introduction of the cases background and objectives, and the online–offline hybrid teaching for the study of relevant theories and methods, students are encouraged to propose their own solutions based on the theories and methods they have learned. Students can design and implement personalized solutions, and in the process, further learn and understand the theories and methods they want to use.

Example of a case: Text recognition of images.

Some X-ray images of welding seam inspection are provided. The goal of this case is to identify all the text on the X-ray images. This is a case with practical engineering needs.

According to the procedure of non-destructive testing (NDT), operators (welding workers) place some leaden markers beside the welding seam. The leaden markers are photographed together with the welding seam. The leaden markers include image quality indicator, positioning markers (center markers, overlap markers) and other identification markers. These identification markers can display the project number, pipe number, welding seam number, welding worker’s ID, welding date, etc.

**Fig. 4:**
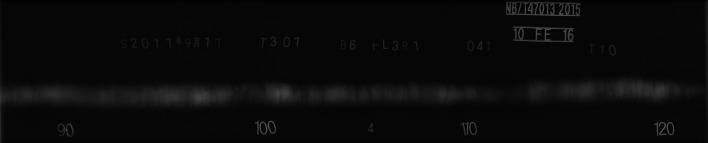
The scanned X-ray imaging film

The welding seam films will be scanned as high-quality digital images by using an industrial X-ray film digitizer. A scanned example image is shown in Fig. 4. The resolution of scanned image is $4242\times 882\times 3$, and the image format is TIFF.

Usually, the solution of this case includes three main parts: image preprocessing, image segmentation, and text recognition. Each part can be implemented in many different methods or a combination of several methods. For example, the methods of image preprocessing include: contrast enhancement, binarization, histogram equalization, geometric transformation, gray level interpolation, noise removal and so on. The methods of image segmentation include: threshold-based segmentation methods (such as Otsu’s method), region-based segmentation methods, and edge-based segmentation methods. In addition, image segmentation may also involve other related technologies, such as Radon transform. The methods of text recognition include: the traditional machine learning methods, such as artificial neural network (ANN), support vector machine (SVM), etc., and the deep learning methods, such as deep convolutional neural network (DCNN), recurrent neural network (RNN), etc. Here, deep learning is the learning technology in the sense of artificial intelligence (AI) rather than the learning method in the sense of education.

**Fig. 5:**
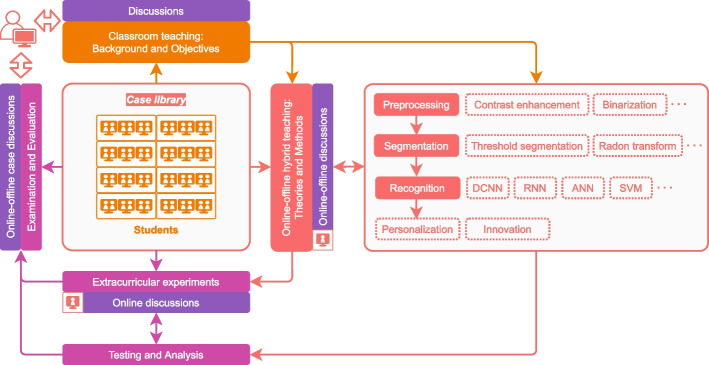
An example (text recognition of images) of the student-centered case-based teaching process

In each part, students can choose one or more methods they want to learn and use according to their own learning ability and learning interest. After making their choice, they need to conduct an in-depth study of these methods. They can download the source codes or write their own codes to implement these methods. Finally, the three parts of the codes are combined to generate their own personalized solution. Because the methods that students choose to learn and use are not the same, the combination of these methods results in a variety of personalized solutions. These solutions need to be tested and evaluated experimentally. Students can communicate any issues they encounter and share their learning experiences through online and offline case discussions. In this process, we encourage students to innovate their own methods or adopt novel ways of combining methods. For this case, the student-centered case-based teaching process is shown in Fig. 5.

### Learning behavior comparison

**Fig. 6:**
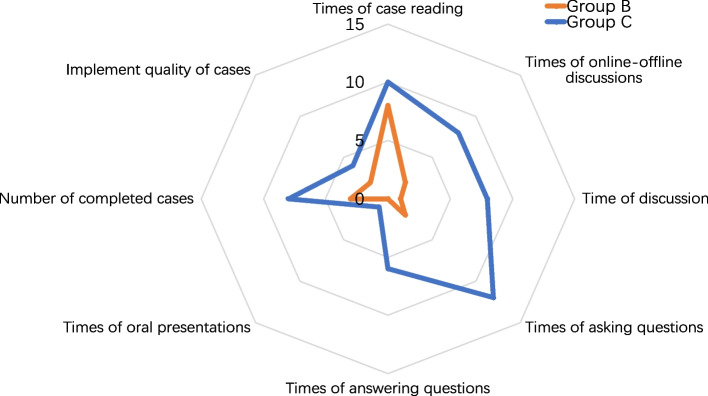
Radar chart of learning behavior comparison

We compared the learning behaviors of Group B and Group C (Group A was the traditional teaching model without providing new cases). Group B adopted extracurricular self-regulated case learning mode. Group C adopted the student-centered case-based teaching and online–offline case discussion mode. According to the statistics, the times of online–offline discussions, the time of discussion, the times of asking questions, and the times of answering questions of Group C students was much larger than that of Group C students. The number of completed cases and the implement quality of cases of Group C students was better than that of Group C students. A radar chart of learning behavior comparison is shown as Fig. 6. The comparison of the two groups of students’ learning behavior shows that the student-centered case-based teaching and online–offline case discussion teaching mode could indeed improve the students’ learning interest and initiative.

### Assessments

In the teaching of Group A, Group B and Group C, Groups A and B adopted the traditional teaching mode. The teaching of Group A did not involve new cases. Students of Group B were provided with case study documents and were arranged for extracurricular self-regulated case learning. Group C adopted the student-centered case-based teaching mode. The assessment methods of three groups were also different. Both Group A and Group B used the traditional examination method. Although students of Group B were arranged to study the case by themselves, the examination contents of Group B did not involve these cases. The assessment methods of Group C included traditional examination, case study reports, experiment reports, etc. In Group B, because the cases are self-regulated learned and the exam did not involve cases, many students did not put a lot of effort into the case study. The examination scores of Group B only improved slightly compared to Group A. The student-centered case-based teaching mode adopted by Group C greatly stimulated students’ learning interest, and their examination scores improved significantly. The comparison results show that the examination scores of Group C are significantly better than those of Group A and Group B. The comparison of the examination scores of the three groups is shown in Fig. 6.

**Fig. 7:**
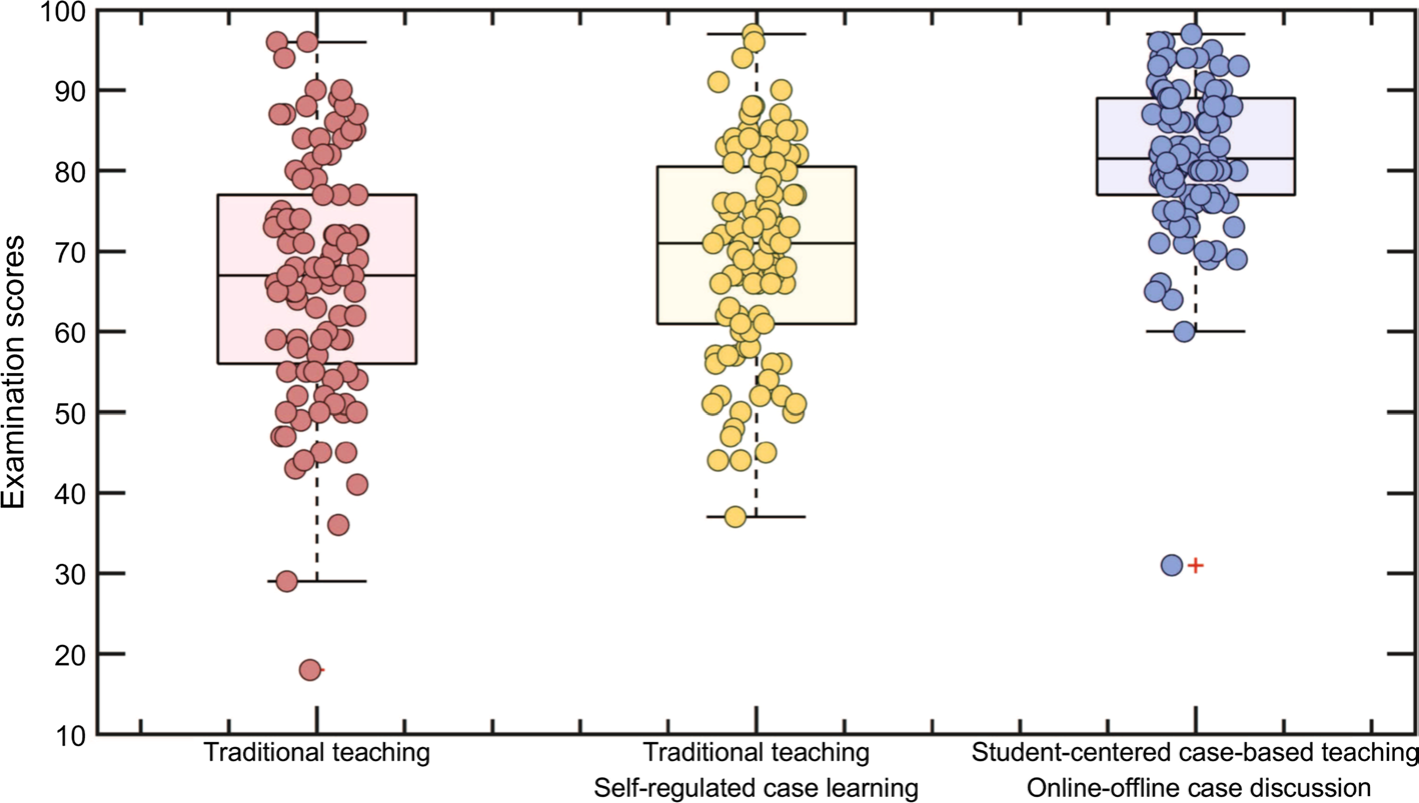
Comparison of examination scores of the three groups

Bloom’s taxonomy can be used as a tool for objective evaluation and examination assessment. It reflects the relationship between knowledge learning and ability development. In the assessments of Group C, we assigned six weights for each examination question according to the revised Bloom’s taxonomy in six aspects: Remember, Understand, Apply, Analyze, Evaluate, and Create. Table 3 is an example of student-centered case-based teaching and online–offline case discussion applying the revised Bloom’s taxonomy. Each cognitive skill corresponds to a specific teaching objective, and these teaching objectives are reflected in the specific test questions.

**Table 3:** An example of student-centered case-based teaching and online–offline case discussion applying the revised Bloom’s taxonomy

Cognitive skills	Description	Example of teaching objectives of text recognition in images
Remember	Recalling basic facts and concepts	Be able to recall relevant knowledge accurately when facing the problem of text recognition
Understand	explaining ideas or concepts	Be able to grasp and understand the various theories and methods used in text recognition
Apply	Using information in new situations	Be able to use the code provided by case library to achieve text recognition
Analyze	Drawing connections between ideas	Be able to distinguish the operation mode and the interaction relationship of each part of text recognition model
Evaluate	Justifying a decision or stance	Be able to judge the accuracy of text recognition model, and find out the existence of errors
Create	Producing a new idea or work	Be able to improve the existing text recognition model, propose new algorithms and models

**Table 4:** *F*-test result of the examination performance

Variable	MS	df	*F*-value	*P*-value	*F* crit
Remember	264.60	1	1.4815	0.2285	4.0069
Understand	1915.35	1	11.1475	0.0015	4.0069
Apply	1215.00	1	9.1703	0.0037	4.0069
Analyze	881.67	1	3.2024	0.0787	4.0069
Evaluate	350.42	1	3.3517	0.0723	4.0069
Create	3270.82	1	13.1502	0.0006	4.0069

We conducted a comparison of the examination performance of students in Group A and Group C according to the revised Bloom’s taxonomy. The test data of the two groups approximately obey the normal distribution and meet the requirement of parameter test. The joint hypotheses test (*F*-test) is used to analyze whether there are significant differences in the knowledge and ability levels of the two groups of students. The *F*-test results are shown in Table 4, where MS represents mean squares, df represents degrees of freedom. Degrees of freedom refers to the number of variables that can be evaluated without restriction when calculating a uniform measure. *F*-value (or *F*-statistic) is the test statistic. *P*-value is the observed significance level. *F* crit represents the *F*-critical value, which is a specific value that *F*-value is compared with. It can be seen from Table 4 that, for Remember, Analyze, and Evaluate, their *F*-values are less than *F* crit and *P*-values are higher than 0.05. This indicates that there is no significant difference between the two groups in these three aspects. For Understand, Apply, and Create, their *F*-values are greater than *F* crit and *P*-value are less than 0.01. This indicates that the two groups of data have very significant differences in these three aspects. The *F*-test results show that, in three aspects of Understand, Apply, and Create, our teaching method has a very significant improvement over the traditional teaching method.

These results can be interpreted as follows. Traditional classroom teaching methods emphasize memorization of basic theories and concepts, based on which students can use these insights to solve problems and pass exams. Therefore, students are fully trained in the three cognitive skills of Remember, Analyze, and Evaluate. In these three aspects, its learning effect is no less than the student-centered case teaching. However, due to the lack of specific application training, traditional classroom teaching methods do not allow students to understand the basic concepts more deeply. Students’ ability of association and innovation has not been fully trained. This is reflected in students’ difficulty in applying what they have learned to solve complex engineering problems.

Student-centered case-based teaching and online–offline case discussion provide students with an environment and opportunity to carry out specific application training, which help students actively explore and understand basic concepts, and apply the knowledge learned to practical engineering problems. Compared with the traditional classroom teaching mode, student-centered case-based teaching mode can improve students’ enthusiasm and initiative in learning, and improve their ability to solve complex engineering problems. This is reflected in the improvement of three cognitive skills: Understand, Apply, and Create.

## Discussion

Digital image processing is a highly theoretical and practical course. When using the case-based teaching method, the physical concepts and meanings behind mathematical formulas should be emphasized in classroom teaching, and the methods and principles should be explained thoroughly. We try to let students truly grasp the theoretical principles and understand engineering applications through the introduction of engineering cases. The main characteristics of our student-centered case-based teaching are as follows.

1. Using cases as the main content of teaching
  At present, the digital image processing teaching materials used in this course cannot fully meet the needs of postgraduate teaching, and we do not find a better alternative textbook. Therefore, changing the main teaching content from textbook to cases is a solution. In our student-centered case-based teaching, cases are the main content of teaching. This method stimulates learning interest of students. Students can deepen their understanding of knowledge in the process of solving engineering problems.
2. Adopting a case-based teaching method
  Knowledge points of the textbook are guided by the needs of engineering applications. The knowledge points are presented to students in the way of interconnected applications, so that students can discover and master knowledge in the practice process of solving-problems.
3. Student-centered teaching design
  Through case-based teaching, the student-centered teaching design is truly realized. The student-centered learning approach not only allows students to choose what to learn, but also to choose how to learn.
4. Online and offline case discussions
  Case discussion promotes knowledge construction through the process of shared exploration. Case discussion is the key to the success of case-based teaching. In the teaching method we designed, the case discussions can be transferred to online, and the real-time synchronous discussions can be transferred to the online asynchronous discussions.

Compared with the traditional teaching mode, the student-centered case-based teaching and online–offline case discussions proposed in this paper have achieved the following improvements in the teaching of postgraduate courses of computer science.

1. Students have a deeper and better understanding of the digital image processing course. The case-based learning model enables personalized learning by using offline-online hybrid approaches, supported by expanded learning options and multiple case resources (Jevne et al., 2021; Yoon et al., 2021). Through case-based teaching, they have fully realized the importance and practicability of this course.
2. Through the student-centered case-based teaching method, the boring knowledge teaching is replaced by the flexible and diverse case teaching, which arouses learning enthusiasm and interest of students. The study found that students can organize their learning process, and students’ time management flexibility and flexibility content are quite high, which were stated in the literature (Endedijk et al., 2016; Turan et al., 2022).
3. Teaching effect is improved. Students’ theoretical level, practical level, ability of analyze and solve problems, innovative thinking mode and literature reading level are improved to a certain extent. This result can be confirmed by the comparison of examination scores of the three groups (Fig. 7) and the learning behavior comparison of the two groups (Fig. 6). Empirical studies on how students learn, including brain development, motivation, creativity, perseverance, self-regulation, knowledge application, etc., also confirm the effectiveness of student-centered learning approaches (Goodell & Thai, 2020).
4. Students’ horizons are broadened. Students can understand the knowledge structure and problem-solving methods of different disciplines and courses, and fully realize the advantages of interdisciplinary learning. In case-based learning, there is a need to relate prior knowledge within and between disciplines to external lived experiences. In the process, students are trained in critical thinking, creative thinking, and problem-solving skills and strategies (Jung, 2013).
5. Teaching level of teachers is improved. After the introduction of the student-centered case-based teaching method, the teaching process is no longer completely based on textbooks. Teachers need to think more about the selection and design of cases. This process deepens teachers’ understanding of the curriculum and improves teachers’ knowledge structure. Student-centered case-based teaching can help teachers update teaching concepts, improve teaching methods, and continuously improve teaching levels in subsequent teaching. In student-centered teaching, teachers need to solve problems in communication between students and teachers so that students can receive correct feedback when they need it. Therefore, the teaching management ability of teachers has also been improved (Yan et al., 2021).

## Conclusion

Graduate students of science and engineering usually focus on engineering applications and practices. It is difficult to achieve teaching goals by completely adopting teacher-centered classroom teaching. Case-based teaching can greatly improve the teaching effect. By constructing a case library and integrating the latest engineering cases into teaching, students can better understand the practical application of theoretical knowledge, and generate strong interest in learning and research. We practice the student-centered case-based teaching and online–offline case discussion in a digital image processing course for graduate students in computer science, and propose an actionable case-based teaching scheme. Case-based teaching is a systematic project. In addition to the construction of a case library and the introduction of cases in teaching, it also involves a series of problems, such as the adjustment of teaching plans and the changes of assessment methods. There are still many aspects of case-based teaching that need to be explored and perfected. Through the verification of actual teaching, the student-centered case-based teaching can stimulate learning enthusiasm and interest of students, and help them to cultivate innovative thinking modes and practical abilities. The joint hypotheses test is used to analyze whether there are significant differences in the knowledge and ability levels of students in different learning modes. The *F*-test results show that, in three aspects (Understand, Apply, and Create), our teaching method shows a very significant improvement over the traditional teaching method.

## Data Availability

The data of this study is not open to the public due to participant privacy.

## Abbreviations

CBT: Case-based teaching
CBL: Case-based learning
ENA: Epistemic network analysis
SRL: Self-regulated learning
COVID-19: Coronavirus disease 2019
PBL: Pproblem based learning
AI: Artificial intelligence
NDT: Non-destructive testing
ANN: Aartificial neural network
SVM: Support vector machine
DCNN: Deep convolutional neural network
RNN: Recurrent neural network
